# Asymmetry of Spinal Segments Mobility in Canoeists and its Relationship with Racing Speed

**DOI:** 10.2478/hukin-2013-0004

**Published:** 2013-03-28

**Authors:** Mateusz Rynkiewicz, Tadeusz Rynkiewicz, Włodzimierz Starosta

**Affiliations:** 1 PW Znak – Test Mateusz Rynkiewicz – www.znaktest.pl., Bogdaniec, Poland.; 2 University School of Physical Education in Poznań – Regional Department of Physical Culture, Gorzów Wlkp. Poland.; 3 College of Physical Education and Tourism in Bialystok, Białystok, Poland

**Keywords:** sports training, spine flexion, health, competitive canoeists

## Abstract

The aim of this study was to determine the extent of asymmetry of spinal segment mobility in canoeists. Moreover, the relationship between this parameter and racing speed was analyzed. The study included 18 canoeists with a mean age of 16.4 years. Mobility of cervical, thoracic and lumbar spine, in sagittal, coronal and transverse planes, was measured with the aid of a tensometric electrogoniometer. The racing speed was based on results achieved during the qualifying competition for the Polish national team. Spinal mobility was measured within two days after the competition. Significant associations were observed between average racing speed and the asymmetry coefficients of the cervical (r=−0.52; p=0.03) and lumbar spinal flexure in the coronal plane (r=0.57; p=0.01). The extent of the asymmetry of the cervical spine flexure in the coronal plane should possibly be reduced, because such asymmetry exerts a negative effect on racing speed. In contrast, canoeist’s training should be oriented towards increasing the asymmetry of the lumbar spine flexure in the coronal plane. However, one should keep in mind that such an approach, although favorable in terms of race performance, could negatively affect the canoeist’s health.

## Introduction

Achieving satisfactory sport results in kayaking requires a significant level of muscular strength (Mann and Kearney, 1980). Therefore, athletes practicing this sport need to develop sufficient strength of the upper body, including the trunk (Tesch, 1983; Fry and Morton, 1991; Fekete and Coach, 1998; Akca and Muniroglu, 2008). Canoeing is a paddling discipline that requires the athletes to paddle on one side of the boat, left or right. Such paddling technique requires an asymmetric position of the body and performance of asymmetric muscular work (Rynkiewicz and Starosta, 2011). The canoeist paddles in a step forward/backward position, kneeling on one leg. During the preparatory phase of the paddling movement an athlete performs rotation/bending of the trunk and subsequently utilizes muscular strength of the trunk to maximally propel the canoe during the active phase of the movement. Usually, bilateral paddling is not practiced during canoeing training; therefore, such long-term specialized training can result in an asymmetric distribution of muscular mass and tone (Ilnicka, 1999; Andreoli et al., 2001; Sanchis-Moysi et al., 2004). Abnormal body gait is one possible consequence of disproportions that can even lead to an asymmetric structure of the skeleton (Cibulka et al., 1998; Tanchev et al., 2000; Sanchis-Moysi et al., 2004). Asymmetrical lifting and twisting have been linked to increased incidence of disc prolapse and low back pain (Andersson 1981; Kelsey et al., 1984). Finite element models predicted that maximum symmetric efforts produce tensile strains in the annulus fibers of 10% but when combined with bending and twisting the strains increase to 20% (Shirazi-Adl et al., 1986).

Additionally, injury and joint overload as well as degenerative changes have been reported in some cases as a consequence of these abnormalities (Omey et al., 2000; Tanchev et al., 2000; Kazunori et al., 2006). This is particularly dangerous in young athletes, especially during the phase of intense development of skeletal structures and muscle tissue growth.

Proper spine function, particularly the range of mobility, plays a vital role in determining one’s strength. Previous studies revealed that sport training affects the functional parameters of a canoeist’s spine, and disproportions of spinal mobility have been observed in canoeists depending whether they paddle on the right or left side of the boat (unpublished results of research). All the above-mentioned malfunctions may lead to numerous negative changes manifested later in life. In addition, previous studies have not revealed whether changes in spinal segment mobility influence sports results, in particular the speed achieved during canoe paddling.

The aim of this study was to reveal the effect of side (left and right) of paddling on spinal segment mobility. Additionally, we assessed whether the level of asymmetry of spinal segment mobility is associated with the speed achieved during canoe racing. We tested the hypotheses that left-paddling and right-paddling canoeists differ in terms of the degree of spinal flexure and that the paddling speed is correlated directly with the level of asymmetry in spinal segment mobility.

## Material and Methods

### Participants

The study included 18 advanced canoeists with a mean age of 16.4 years (±SD). Ten athletes were left-sided paddlers and the remaining eight were right-sided. The participants of our study had a very high level of athletic abilities, confirmed by the fact that five of them qualified for the national junior team.

### Procedures

The racing speed, km/h, of the canoeist-canoe system (V) was based on a competition, where Olympic distances were used, held for qualifications to the junior national team. This competition took place two days before spinal segment mobility was measured. Spinal mobility was determined by the electrogoniometric method using a Penny & Giles electrogoniometer (Biometrics Ltd, Gwent, UK) that took measured angular movements in individual spinal articulations (Troke and Moore, 1995; Thoumie et al., 1998; Christensen, 1999; Lewandowski, 2006). This method is characterized by high reliability and precision, and the obtained results are comparable to those determined radiologically and to Polish population normative values (Lewandowski, 2006).

The measurements were taken in cervical, thoracic and lumbar spinal segments. Spinal mobility was determined in coronal, sagittal, and transverse planes, and the respective asymmetry coefficients were calculated based on the following formula (Siniarska and Sarna, 1980):

$$A=\frac{X_{p}-X_{l}}{\frac{\left(X_{p}+X_{l}\right)}{2}}*100\%$$

A – asymmetry coefficient;
X_p_ – the value of a given characteristic determined on the right side;
X_l_ – the value of a given characteristic determined on the left side.

Direct values of asymmetry coefficients (Am) were calculated for the mobility of individual spinal segments, and coefficients of correlation were calculated between those parameters and the paddling speed. This method enabled us to analyze the potential associations between the degree of asymmetry and the racing speed, irrespective of the side of the boat chosen by the canoeists for paddling.

All the procedures of this study were approved by the Local Ethics Committee by the Karol Marcinkowski University of Medical Sciences in Poznan, Poland.

### Analysis

All calculations were carried out using the Statistica 9.0 package (StatSoft, Inc. 1984, 2011, license no. AXAP012D837210AR-7). The results were presented as arithmetic means (M), ± standard deviations (± SD), and the normality of their distributions was verified. Mean values of analyzed parameters determined in athletes paddling on the right and left side of a canoe were compared using ANOVA. Post-hoc tests were used for detailed comparisons of parameters with normal distributions. Due to high variability in the sample size of canoeists paddling on the right or the left side, the Tukey test for unequal samples was used as a post-hoc test. The Kruskal-Wallis test was used for comparisons of variables with non-normal distribution. Additionally, Pearson’s and Spearman’s coefficients of correlation were calculated between the asymmetry coefficients and paddling speed. Statistical significance was defined as p<0.05.

## Results

No significant differences were observed between mean V of right- and left-paddling athletes (Table 1). The only observed significant difference in spinal mobility pertained to the maximal left rotation of the cervical spine (CTL): it was lower in right-sided paddlers (RP) than in left-sided paddlers (LP), 60.38 and 67.7, respectively, for RP and LP left side of the canoe.

The asymmetry coefficients of spinal mobility were subjected to correlation analysis, separately for RP and LP (Table 1). For RP, increasing left rotation of the cervical spine was associated with higher V (r=0.81; p=0.01).

**Figure 1:**
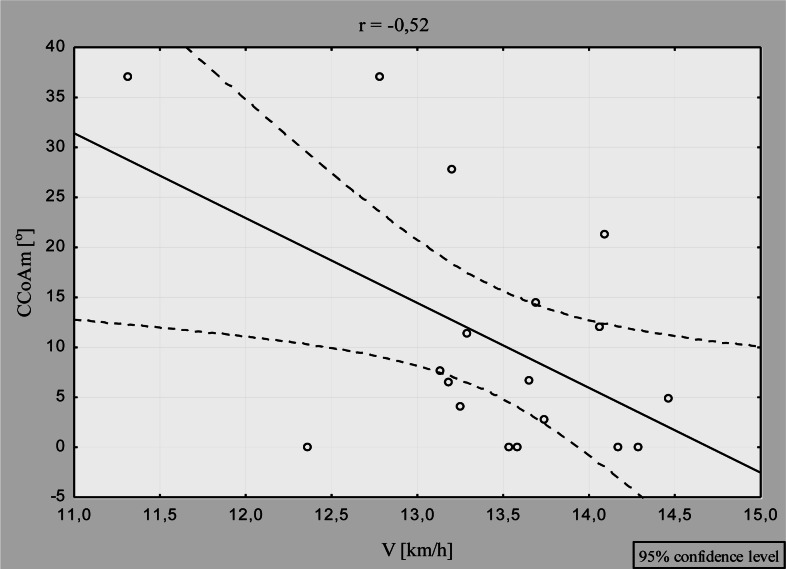
Correlations between paddling speed and asymmetry of the cervical spine mobility in the coronal plane (n=18)

**Figure 2:**
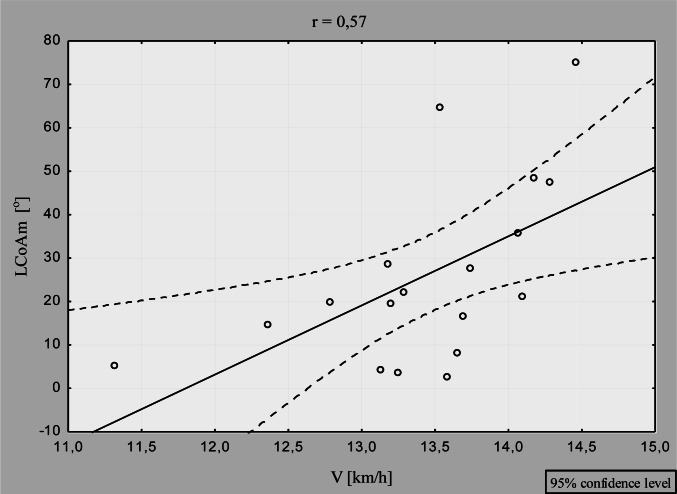
Correlations between paddling speed and asymmetry of the lumbar spine mobility in the coronal plane (n=18)

In the analysis that combined data for RP and LP, significant inverse correlation was observed between the asymmetry of cervical spine mobility in the coronal plane (CCoAm) and V (r =−0.52; p=0.03), (Figure 1). Additionally, significant direct correlation was revealed between the asymmetry of lumbar spine flexure in the coronal plane (LCoAm) and V (r=0.59; p=0.01) (Figure 2).

## Discussion

Our study revealed that the degree of left rotation is significantly lower in RP as compared to LP. Higher degree of left rotation documented in canoeists preferring the left side could be associated with the specificity of canoe paddling. Torsion of the body is required to achieve propulsion; during this movement the head is frequently rotated to the opposite side and kept parallel to the direction of canoe movement (Rynkiewicz and Starosta, 2011). In such cases, left rotation of the cervical spine is observed in athletes paddling on the left side of the canoe, whereas right rotation takes place in right-sided canoeists. A lack of similar intergroup differences in the right rotation of cervical spine can result from individual characteristics of head position during paddling.

Correlation analysis revealed an interesting finding: an increase in the left rotation of cervical spine was found to be associated with a higher racing speed in RP. This correlation suggests that the degree of head rotation is similar to that of the trunk, and consequently that the head is not kept parallel to the direction of canoe movement as previously suggested. Rotating the head in the direction of canoe movement can lead to rigidity of the cervical spine, preventing its contralateral rotation and blocking the development of significant strength of the spinal extensors. Consequently, one can assume that athletes with greater left rotation paddle with their heads positioned in the same direction as their trunks. As a consequence of long-term sports training, a higher mobility of the cervical spine is achieved and a relatively better racing speed is accomplished as compared to athletes with lesser rotation.

Furthermore, we observed that the level of asymmetry in the mobility of the cervical spine in the coronal plane was inversely correlated with racing speed. This means that an increase in the asymmetry can be reflected in lower speed (Figure 1). The level of asymmetry was higher in RP, but this difference proved insignificant due to high variability of individual results. The asymmetry observed in the cervical spine mobility in the coronal plane resulted from the asymmetry of sport technique. This negative tendency can lead to degenerative changes within the spine, having harmful health consequences (Sward, 1990; Sward et al., 1990; Cibulka et al., 1998; Omey et al., 2000; Tanchev et al., 2000; Sanchis-Moysi et al., 2004; Kazunori et al., 2006).

In contrast, we observed a significant positive correlation between the asymmetry of the lumbar spine flexure in the coronal plane and racing speed. Specifically, increased asymmetry was associated with a higher racing speed (Figure 2). Many years of sport’s training are reflected by adaptive changes, including asymmetry of mobility of spinal segments. These types of adaptive changes are necessary to increase racing speed; however, it is still unclear whether the resulting changes affect an athlete’s health negatively. Long-term training is reflected by the asymmetry in the distribution of skeletal structure (Sanchis-Moysi et al., 2004), muscle mass and its tone (Cibulka et al., 1998; Ilnicka, 1999; Andreoli et al., 2001) as well as spine mobility, potentially leading to injuries and degenerative changes (Andersson, 1981; Kelsey et al., 1984; Sward, 1990; Sward et al., 1990; Omey et al., 2000; Kazunori et al., 2006). Resulting changes in the spine are particularly harmful to athletes’ health and can limit their normal functioning in everyday life after finishing their professional careers (Picture 1).

## Conclusions

A comparison of athletes paddling on the right and left side of the canoe revealed significant differences in the degree of left rotation of the cervical spine in the transverse plane.

Increased asymmetry in the cervical spine flexure in the coronal plane negatively influences racing speed. In contrast, higher asymmetry of the lumbar spine flexure in the coronal plane was associated with higher values of paddling speed.

## Figures and Tables

**Picture 1:**
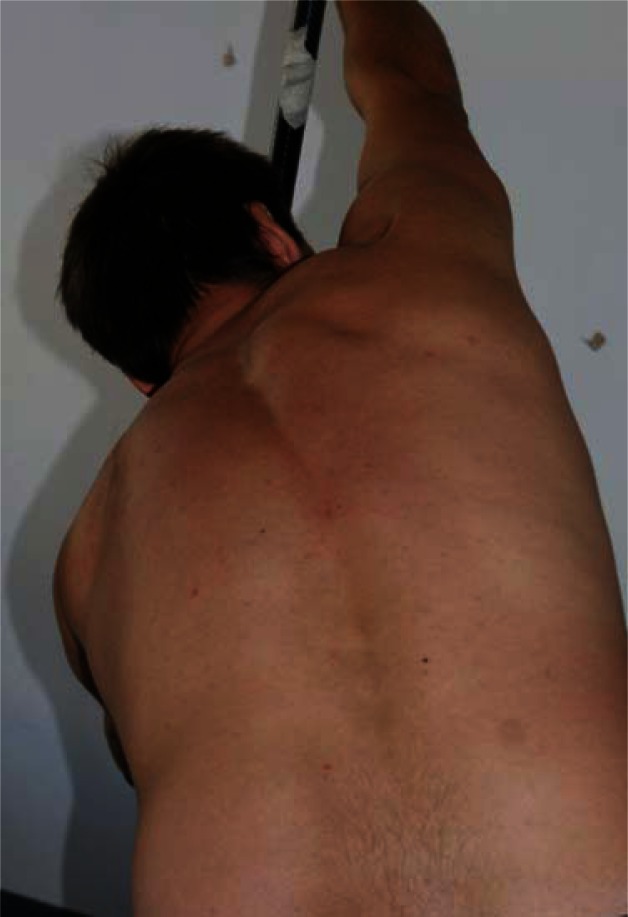
Back view of the left side paddler in the phase of water grip

**Table 1:** Anthropometric measurements and coefficients of asymmetry in right side (RP) and left side (LP) paddlers (n=18)

	**RP n=8**		**LP n=10**	
	**M**	**± sd**	**M**	**± sd**
V [km/h]	13,45	1,05	13,42	0,47
CSA [%]	−73,86	37,43	−73,65	24,21
CSAm [%]	75,29	34,03	73,65	24,21
CCoA [%]	−6,98	23,12	−1,69	7,45
CCoAm [%] *	**17,46**	**15,55**	**5,45**	**5,07**
CTA [%]	2,40	19,16	−7,79	18,61
CTAm [%]	26,00	6,61	31,50	11,42
ThSA [%]	−72,36	60,81	−50,79	54,39
ThSAm [%]	73,97	58,55	62,40	38,70
ThCoA [%]	−5,31	24,94	4,33	28,64
ThCoAm [%]	19,42	14,93	23,45	15,16
ThTA [%]	2,47	60,29	0,27	52,10
ThTAm [%]	51,48	24,76	41,06	28,99
LSA [%]	116,27	34,20	84,32	30,78
LSAm [%]	116,27	34,20	84,32	30,78
LCoA [%]	−5,09	41,08	1,56	29,13
LCoAm [%] *	**32,47**	**22,63**	**20,75**	**19,30**
LTA [%]	18,36	70,60	7,19	40,98
LTAm [%]	56,49	41,52	33,43	22,24

* Significant correlation between speed and spinal mobility factor

M, mean; ± SD, standard deviations; C, cervical spine; Th, thoracic spine; L, lumbar spine; Co, coronal plane; S, sagittal plane; T, transverse plane; A, asymmetry; Am direct values of asymmetry

## References

[b1-jhk-36-37] Akca F, Muniroglu S (2008). Anthropometric – Somatotype and Strength Profiles and On – Water Performance in Turkish Elite Kayakers. International Journal of Applied Sports Sciences.

[b2-jhk-36-37] Andersson GBJ (1981). Epidemiologic aspects on low-back pain in industry. Spine.

[b3-jhk-36-37] Andreoli A, Monteleone M, Van Loan M, Promenzio L, Tarantino U, De Lorenzo A (2001). Effects of different sports on bone density and muscle mass in highly trained athletes. Med Sci Sports Exerc.

[b4-jhk-36-37] Christensen HW (1999). Precision and accuracy of an electrogoniometer. J manip physiol ther.

[b5-jhk-36-37] Cibulka MT, Sinacore DR, Cromer GS, Delitto A (1998). Unilateral hip rotation range of motion asymmetry in patients with sacroiliac joint regional pain. Spine.

[b6-jhk-36-37] Fry RW, Morton AR (1991). Physiological and kinanthropometric attributes of elite flatwater kayakists. Med Sci Sports Exerc.

[b7-jhk-36-37] Ilnicka L (2005). Variability of selected macroscopic properties of muscles and their relationships and the overall structure of the human body. (From studies of individuals living anthropomorphological).

[b8-jhk-36-37] Kazunori I, Koichi N, Kazunori I, Hideo F, Hiroyuki N (2006). Low back pain and lumbar disc degeneration are related to weight category in collegiate wrestlers. Med Sci Sports Exerc.

[b9-jhk-36-37] Kelsey JL, Githens PB, White AA, Holford TR, Walter SD, Conner T, Ostfeld AM, Weil U, Southwick WO, Calogero JA (1984). An epidemiologic study of lifting and twisting on the hob and risk for acute prolapsed lumbar intervertebral disc. J Orthop Res.

[b10-jhk-36-37] Lewandowski J (2006). Evolution of physiological curvatures and ranges of human spine segmental mobility at the age of 3–25 years in the image of electrogoniometric method.

[b11-jhk-36-37] Mann R, Kearney J (1980). A biomechanical analysis of the Olympic-style flatwater kayak stroke. Med Sci Sports Exerc.

[b12-jhk-36-37] Omey ML, Micheli LJ, Gerbino PG (2000). Idiopathic Scoliosis and Spondylolysis in the Female Athlete. Clin Orthop Relat R.

[b13-jhk-36-37] Rynkiewicz M, Starosta W (2011). Asymmetry of paddling technique, its selected conditions and changeability in highly advanced kayakers.

[b14-jhk-36-37] Sanchis-Moysi J, Dorado C, Vicente-Rodriguez G, Milutinovic L, Garces GL, Calbet JAL (2004). Inter-arm asymmetry in bone mineral content and bone area in postmenopausal recreational tennis players. Maturitas.

[b15-jhk-36-37] Shirazi-Adl A, Ahmed AM, Shrivatava SC (1986). Mechanical response of a lumbar motion segment in axial torque alone and combined with compression. Spine.

[b16-jhk-36-37] Siniarska A, Sarna J (1980). Asymmetry of human body – a synthetic approach. Studies in Human Ecology.

[b17-jhk-36-37] Sward L, Ericksson B, Peterson L (1990). Anthropometric characteristics, passive hip flexion and spinal mobility in relation to back pain in athletes. Spine.

[b18-jhk-36-37] Sward L (1990). The back of the Young top athlete: symptoms, muscle strength, mobility, anthropometric and radiological findings.

[b19-jhk-36-37] Tanchev PI, Dzherov AD, Parushev AD, Dikov DM, Todorov MB (2000). Scoliosis in Rhythmic Gymnasts. Spine.

[b20-jhk-36-37] Tesch PA (1980). Physiological characteristics of elite kayak paddlers. Can J Appl Sport Sci.

[b21-jhk-36-37] Thoumie F, Drape JL, Aymard C, Bedoiseau M (1998). Effects of a lumbar support on spine posture and motion assessed by electrogoniometer and continuous recording. Clinical Biomechanics.

[b22-jhk-36-37] Troke M, Moore AP (1995). The development of a new form of instrument fixation for the OSI CA 6000 spine motion analyzer. Manual Ther.

